# Personalized commissioning, public spaces: the limits of the market in English social care services

**DOI:** 10.1186/1472-6963-13-S1-S5

**Published:** 2013-05-24

**Authors:** Catherine Needham

**Affiliations:** 1 Health Services Management Centre, University of Birmingham, UK

## Abstract

**Background:**

The article explores the implications of personal budgets within English social care services, which position the individual as market actor. Rooting the research in the broader personalization agenda, the study looks at the limitations of the market in relation to individual purchase of private goods (e.g. home care), in the pooling of funds to purchase group services and in the provision of public goods such as building-based services.

**Method:**

The article takes a multi-method approach, combining an interpretive focus on the framing of the personal budget-holder by advocates of personalization with national evaluation data, and data from a small survey of day centre workers.

**Results:**

The article identifies three framings of the individual budget-holder articulated by advocates of personalization. The first is that personal budget-holders will be empowered market actors, commissioning the services they need. The second is that budget-holders will pool resources with others to purchase group services in order to broaden the range of options available to them. The third is that services which cannot be disaggregated into individual or group budgets – such as day centres – are not valued by service users. The article looks at the evaluation data on these three claims in turn. It identifies four limitations to the capacity of people to purchase care goods on an individual basis: lack of transparency in allocating budgets, complexity in managing a budget, excessive auditing of spending and lack of responsiveness from the provider market. Pooling of budgets to purchase collective services is found to be underdeveloped, and hampered by the complexity which is a broader limitation on personal budgets. Day centres are found to be closing not in response to commissioning decisions by individual budget-holders but because of *decommissioning* by local authorities, minimising the scope for individuals to express a preference for this type of care. The survey highlights patterns of day centre closure, rising fees for attendance and reduced eligibility, and the underdevelopment of mechanisms to facilitate commissioning of new collective spaces.

**Conclusions:**

The paper concludes that the transition to personal budgets – in the context of the accompanying financial crisis in local authorities – has led to inadequate attention to the potential for an undersupply of collective and public goods. The loss of day centre provision will be felt by personal budget holders but also by self-funders and people in residential accommodation who may no longer be eligible for, or able to afford, to access shared spaces. Local authorities are actively taking on the role of decommissioners without sufficient responsiveness to how and what individuals want them to commission.

## Introduction

The disaggregation of care spending into personal budgets for eligible users in English social services is creating a set of new market actors, making purchasing decisions about their care. Whereas commissioning has historically been an activity undertaken by local authorities on behalf of service users, individual budget-holders will now act as commissioners, deciding how to spend their allocation. This article explores how these individual commissioners are likely to interact with the market and what are some of the limitations in what the market can offer the care recipient as commissioner.

Before looking at the detail of new markets in care services it is important to be aware of the changes which have been undertaken in English social care services under the umbrella heading of personalization. The first section below provides some brief contextual information (for more details on the background of personalization see [1] or [2]). The article then goes on to look at how the move to personal budgets has framed the individual market actor. Taking an interpretive approach to the claims made by personalization advocates, it highlights three elements to the personalization ‘story-line’ as it pertains to the individual as commissioner. The first is that personal budget-holders will be empowered market actors, commissioning the services they need. The second is that budget-holders will pool resources with others to purchase group services in order to broaden the range of options available to them. The third is that services which cannot be disaggregated into individual or group budgets – such as day centres – are not valued by service users.

The sections that follow use existing evaluation data on personalization, along with a survey conducted by the author, to interrogate these claims. The limitations of the budget-holder as a commissioner of personal care services is considered, before going on to look at the limitations of group and collective commissioning in a context of personal budgets. Alongside the existing evaluation data, the article draws on new survey data to look at how far day centres, as an example of a collective good, are viable in a context in which individuals are increasingly commissioning their own services.

## Personalization and personal budgets

English social care services are undergoing a period of profound transformation triggered by the personalization agenda. Local authorities were given funds of £520 million to redevelop their provision according to personalized principles: to increase choice and control, improve prevention, enhance social capital and improve access to universal services [3]. All people who qualify for means- and needs-tested financial support from the local authority to cover the cost of care (primarily older people, people with disabilities and some carers) must be moved to a personal budget as quickly as possible. The budget can be taken as cash (a ‘direct payment’), as a budget managed on their behalf by the local authority or a third party, or as a combination of the two [1].

Direct payments for care recipients are not new. Within the social care sector initiatives to transfer resources to disabled people as direct payments began in the 1980s, and were given legal endorsement in the 1996 Community Care (Direct Payments) Act. Direct payments were generally taken up by younger disabled people, and numbers were relatively small [1]. What is new is the scale of the expansion, and the introduction of managed personal budgets so that people who do not want to handle the money themselves will have greater choice and control over care services. A survey of local authorities in 2012 found that around 430,000 of people were on a personal budget (53 per cent of those eligible), an increase of around 40 per cent in one year [4].

With the move to personal budgets, the previous practice, through which local authorities provided services themselves or commissioned them from external providers through long-term block contracts is shifting. It is now service users who are expected to be commissioners, purchasing services directly from providers as direct payment holders or working with local authorities to arrange spot purchasing. The role of local authorities is shifting to being ‘market shapers’ rather than large-scale commissioners of services [5].

## Three framings of the individual commissioner

The work of interpretive policy analysts such as Hajer [6], Fischer [7] and Yanow [8,9], has drawn attention to the ways in which policies function as stories that are told about social reality. A dominant story-line provides a compelling account of a policy which ‘sounds right’, based on its plausibility, trust of the author, and acceptability for the listener’s own discursive identity [6]. Elsewhere I have argued that there is a personalization narrative [2], extracted from claims made by advocates of personalization, for example in publications from the Department of Health, the Association of Directors of Adult Social Services (ADASS), the social enterprise In Control and the Social Care Institute for Excellence (SCIE), by social entrepreneurs (e.g. [10,11]) and broadly sympathetic academics (e.g. [1]). These texts broadly share an account of personalization, such that they constitute a ‘communit[y] of interpretation’ [9]. This article uses the same data to analyse the stories which this interpretive community tell about the role of the individual as commissioner within a context of personal budget-holding. Three themes emerged:

(1) Having a personal budget will empower people as individual commissioners

There is an optimism amongst personalization advocates about the extent to which being a commissioner will empower people. Control over the money has been identified as the crucial dynamic in achieving better outcomes. A member of the social innovation network In Control told Needham, in an interview, ‘Until I’ve got hold of the money, or at least I’m directing the way that that money’s spent, that provider is never going to listen to me. It’s the power of the pound, the power of having the money is the bit that makes the difference’ [2][p.55]. Control of the ‘purchasing power’ is expected to drive market responsiveness [12][p.8].

Financial control is expected to deliver a range of benefits alongside the broader aim of better outcomes. It is seen as enhancing personal efficacy, contributing to ‘self-confidence, morale and emotional and psychological health in a range of areas’ [1, pp.117-8]. It is expected to facilitate inclusion in the broader citizenry, enabling people to participate in the rights and duties of citizenship [13]. There is perceived to be a normalisation process associated with financial control, in which people become full members of the citizenry, accessing universal services, rather than people segregated into special purpose sites and services [14].

(2) People will be able to pool their budgets to become group commissioners of services

Alongside personalized commissioning of care, advocates of personalization have anticipated group pooling of funds, in which people come together to make shared purchasing decisions. As Andrew Tyson from In Control puts it,

*What commissioners and others in the local authority will sometimes need to do is to open doors for people who have previously relied on special services. For example a group of friends who are users or former users of learning difficulty day services may want to pool money from their personal budgets to rent a room in community centre for a party or a meeting or to hire a five a side football pitch or a coach to go on a trip. They might need someone to talk to the leisure centre*, *community centre*, *or the coach company for them. There are no doubt a number of ways to achieve this*, *but one of them is for them to put their money in a pot and ask for assistance to find a support worker to do these tasks for them. *[12][p.21]

The Government’s *Vision for Adult Social Care* called for greater pooling of care budgets to ‘allow the focus to shift away from funding streams and onto people’s needs’ [15][p.24].

(3) Collective commissioning of building-based services is not required because these are not valued services

Pooling of budgets can potentially support distinctive groups of budget-holders. However a separate issue is the extent to which it is possible to supply public spaces – accessible to all community members, albeit on the basis of some eligibility criteria – in a context of personal budgets. Building-based services such as day centres constitute such public goods, in the sense that they are not funded by groups of individuals pooling their funds but are accessible shared spaces which are collectively provided. It is not easy to see how such services can be provided in a context of personal budgets.

The response of many personalization advocates to people’s concerns about day centre closures is that these are not services that people value and that their demise will be a welcome effect of transferring purchasing decisions to individuals. Day centres are framed as dreary, unfulfilling sites for the warehousing of older and disabled people [10,11,16]. Duffy is scathing of the claims that subsidies should continue to be provided to ‘…[c]ertain kinds of congregate provision…because they won’t survive if people have choice about the services they use – this seems a weak argument, particularly given that the rationale for such services is their supposed “efficiency”’ [17][p.21]. There is optimism about the new collective enterprises which personalization may facilitate. As Keohane puts it, ‘...personalization does not necessarily mean individualising. It may mean enabling individuals to collect together and form new organic collectives’ [18][p.47].

## Findings

These three claims about how the market will respond to the individual commissioner can be looked at in turn. The discussion here draws on existing evaluative data on personalization, including data from the National Personal Budget survey conducted by Think Local, Act Personal (TLAP), Lancaster University and In Control, from the Association of Directors of Adult Social Services (ADASS), from DH-funded evaluations such as Glendinning [19], and from meta-evaluations of personalization studies such as Glasby and Littlechild [1]. However all of this data has been subject to challenge, for example because it is funded by organisations with a vested interest in promoting the personalization agenda, or because it measures the experience of people who took up a personal budget at an early stage who are likely to be atypical of the majority of social care service users (see for example [20]).

### Individuals as commissioners

A recurrent finding from the evaluations is that the people who have the greatest amount of financial control – those on direct payments – have the most positive outcomes. The National Personal Budget survey conducted by Think Local, Act Personal (TLAP), Lancaster University and In Control, concluded: ‘Whilst all personal budget holders reported positive outcomes, those managing the budget themselves as a direct payment reported significantly more positive outcomes than people receiving council managed budgets’ [21][p.4]. Being a direct employer was seen as a key element in explaining higher satisfaction rates for direct payment holders. People who employed their personal assistants directly rather than through a care agency got better continuity, greater control and an enhanced quality of life [22,23].

Their greater perceived effectiveness is the reason why the Department of Health (DH) and TLAP (a coalition of groups supporting personalization) specify that direct payments should be the default option for personal budget holders. However a number of studies have highlighted limitations in the extent to which direct payment holders can command market power. First, concerns have been expressed about the extent to which people are not made to feel that they have control over the money, either because of excessive restrictions on what can be purchased (limiting choices to a pre-determined menu), intrusive auditing procedures or the ‘clawing back’ of unspent funds at the end of the year [24]. Second, there has been concern that the transparency which was supposed to be a key element of the move to personal budgets has been compromised by the adoption of opaque Resource Allocation Systems (RAS) to calculate budget entitlements. Analysis of RAS used in 20 local authorities found: ‘No local authority contacted by the authors appeared geared up to share the inner workings of their RASs with service users, or to be able to explain in clear and simple terms their underpinning assumptions...’ They concluded, ‘far from leading to more transparent, fair and equitable allocation of resources, RASs obfuscate discretionary care planning processes and make it harder for service users to challenge unfair or inequitable allocations’ [25].

Third, the complexity of managing a direct payment has been a significant problem for many users. The National Personal Budget survey found ‘The single most commonly commented upon issue in the survey was a lack of clarity, often regarding how money could or couldn’t be used, but also concerning other aspects of personal budgets as well’ [21] [p.19]. An evaluation of personal budget take-up in Essex, reported, ‘Service users and family members explained that while in many cases frontline staff appeared confident in selling the initial idea of cash payments, they felt they often struggled to explain the “nuts and bolts” of how they work’ [26][pp.7-8]. There are equality issues relating to citizen’s ability to navigate these complex systems, with some people being more likely to utilize cash resources more effectively than others [19,27]. Indeed there has been a suggestion that the better outcomes recorded for people with direct payments over managed budgets are due to the tendency of well supported and resourced people to opt for direct payments in the first place [20,28].

Fourth, there are indications that the provider market is not yet responding to the purchasing power of the individual. For example, there are doubts about how far local labour markets are providing suitably skilled and competent personal assistants for employment by direct payment holders [19] [p.457]. There are also concerns about the extent to which local authorities are shifting quickly enough to their new role as market shaper rather than commissioner of services [5]. Micro-providers (those with 5 or fewer staff), who the Department of Health see as key to delivery of personalized care services, are particularly struggling with the regulatory burden that local authorities impose [29]. To become a ‘preferred provider’, which local authorities will recommend to personal budget holders, often requires compliance with procurement procedures that are beyond the capacity of very small organisations.

Some of these concerns may ease as direct payment becomes more of a mainstream activity and people share ideas on how to manage complexity and identify good quality providers. However the well-established self-funder market for home care services provides a useful insight into the practice of individuals as long-term commissioners of care. Forty per cent of older people are estimated to make some financial contribution to their care costs [30]. However according to an evaluation of self-funders, ‘For some people there was a profound sense of “powerlessness” and lack of control over their own financial resources, coupled with some real fear over what would become of them if their savings ran dry...’ [31][p.50]. The poor quality of home care services – described by the Equalities and Human Rights Commission [32][p.7] as constituting ‘serious, systemic threats to the basic human rights of older people’– attests to the weakness of individual purchasers in the home care market, and the need for system-level action on commodification rather than over-optimism about the ‘purchasing power’ of individuals.

### Budget-holders as group commissioners

The second story-line is that people will be able to combine with others to commission group services. Existing evaluative data indicates that some pooling of resources is occurring. A National Audit Office review of personal budgets noted: ‘We also found many examples of users finding innovative ways to use their budget to achieve care outcomes, for example, by pooling their budgets to pay for a personal assistant to help with care needs’ [33][p.7]. However, there are clear limitations to the scope which people currently have to recollectivise their budgets, including the practical difficulties of making pooled budgets work in a range of settings. Staff may think that personal budgets are ‘not allowed’ to be pooled [34]. In an evaluation of pooled budgets by the charity HACT, the pooling of budgets was recognised to be impeded by internal systems and processes including staff contracts, disaggregation of services, individual pricing and back office IT and finance systems [35]. In one case gym equipment had been purchased collectively by people in a supported housing facility but use of it was being slowed up by uncertainties about who should have access and what happens when one of the original purchasers leaves the facility [35].

### Collective commissioning

The third claim is that collective commissioning of public goods such as building-based services is no longer required as people make different choices through individual and group commissioning. To gain insight into the extent to which individual commissioning decisions are triggering changes to day centre provision, the author conducted a small survey of staff working in day centres. The email survey was circulated by Unison, the public services union, to its day centre members. It was sent out in January and February 2012 to 200 Unison branches and had 123 responses. Given that there are no reliable national figures on how many staff work in day centres across England, and that it is likely that respondents who are unionised and concerned about day centre closures were more likely to complete the survey, it is not presented here as a representative sample; the need for further work to build a representative national picture is fully appreciated. Brief descriptive statistics were extracted from the survey, and are discussed below. However the analysis focused on verbatims from the survey, which highlight some of the changes in practice surrounding day centres. Verbatims were coded by the author in a two-stage process. The first stage involved the author reading and re-reading the transcripts in order to develop and refine codes and categories. The second stage involved comparing the emerging themes across the transcripts in an iterative process to ensure that all the data were accounted for and to identify convergent and divergent themes.

Day centre workers who responded to the survey indicated that day services in their areas are changing, either through centre closures, changes in eligibility criteria or changes in charging structures. In the survey, over half (56 per cent) of respondents reported that day centres have closed in their area in the last three years. Half of those aware of closures gave the number of day centres affected, with a median number of closures of two.

The story-line around day centre closures identified above is that day centres will close because they do not conform to the preferences of individual budget-holders. However in the closure cases reported in the survey, consultation of service users was often minimal. Respondents were asked what consultation was undertaken by the local authority prior to closure or redesign of day centre services. In those areas where closures have taken place, 59 per cent of respondents indicated that the local authority did undertake consultation (for at least one centre). Of those giving detail on the consultation, the procedures were widely perceived to be inadequate either because the consultation period was too short, responses were ignored and/or insufficient attention was given to what people would do in the absence of the day centre.

*“Council has cut all older peoples day centres. Gave the least amount of consultation as they could get away with*, *no consideration was given to what the older people would do when the day centres closed.”*

*“We were consulted*, *however questions asked/options were designed in such a way that retaining current services was not an option. Also some vital questions were not asked. When asking about engaging in the community the ‘wish list’ did not take into account the cuts in the third sector funding and cuts to peoples benefits.”*

Respondents were asked about the impact of personal budgets on day centre provision. It was clear that many people saw a close link between the expansion of personal budgets and the reduction in day centre facilities:

“Council say self-directed support clients are not choosing to attend day centres as they want to do other things.”

“There is generally a thought that service users could reconsider how day care is provided by using the money in their budgets to provide what they want.”

“Some older people and people with learning disabilities are choosing to create a package of support that doesn't involve day services.”

“This £30 [daily budget] can also be used by service users to access community resources and has been used to provide carers to enable someone to get out of the house.”

Some respondents were optimistic about the scope for user choice that personal budgets could provide:

*“More support for family carers*, *looks at the whole life of the customer*, *not just day service needs.”*

However many respondents expressed concerns about the viability of personal budgets in maintaining the quality and scope of services. Concerns included:

• Personal budgets that are too small to cover care needs

“Budgets for personal use were reduced or did not cover anything like previous provision as the alternative services are more expensive to purchase. Brokering was limited. A significant amount of users were left with no service at all.”

“Peoples budgets are not as big as they thought and once basic care needs are met there is often not alot left for what I would class as social care. The result is more people pulling out of services and becoming more isolated. They are also becoming more reliant on the few carers they see to provide a complete service in a few hours.”

“People who have now been assessed for a personal budget can come out with less money for day services so may not be able to afford amount of previous day opportunities.”

*“PBs do not cover the cost of a full day's attendance for many*, *and this fragments provision and makes it more difficult to cater for.”*

• Inadequate choice for people with personal budgets

“those on personal budgets [are] struggling to find activities.”

*“[We] started to provide group activities within day service that could be 'bought' by those on individual budgets*, *and supported by PAs. Now this has been closed. No one can buy individual sessions anymore.”*

• Insufficient funding for building-based services

“it remains unclear as to who will fund the rent to these community centres.”

*“Trips have had to be axed*, *we don’t have personal budgets to give birthday presents*, *biscuits in the morning as some people attending won’t have had a breakfast or a hot drink or drink before attending.”*

When a centre closed or was redeveloped, some respondents reported positive outcomes, indicating that more ‘personalized’, community-based services or hubs were now available, in place of the day centre:

“Physical disability services changed from traditional day centre provision to a 'personalized' service - service users signposted to community activities/ groups etc and a weekly drop in service.”

“5 Learning Disability centres are now multifunctional centres shared with leisure and cultural facilities. Centres increasingly used as a hub with more activities outwith.”

However for others the transition to new services had not been a satisfactory one:

“Users were ‘signposted’ to alternatives which were basic and much reduced when they were given any alternatives.”

“No checking with alternative day centres to see if places were available and if the same amount of days were possible”

*“Day centres for older people were closed at their original place*, *and all people moved long distances to makeshift day centres.”*

*“Learning disabilities*, *physical disabilities*, *elderly day centres clumped together at mealtimes*, *older people cannot adjust to the noise or the lack of attention to their various health issues.. The day service centres that are now used are inadequate for older people*, *too cold*, *no proper provision for constant heat during the cold spells*, *too long to travel to the centres for many.”*

*“The expectation is that they will be supported from home - even if they have a minimal amount of hours. Only those classed as complex*, *severe or at risk are to receive a day service.”*

Thirty per cent of those experiencing closures stated that no alternative provision was made for at least some service users, whilst a further 20 per cent reported that they didn’t know if any alternative was available.

Alongside day centre closures it was evident from the survey that many centres are changing the service that they provide. 71 per cent of respondents had noticed changes to day centre provision in the last three years (aside from centre closures). Changes included restricting eligibility and introducing charges for users. Two-thirds of survey respondents indicated that restrictions were being placed on the eligibility of service users accessing day centres. This included:

• Reducing the number of sessions that people could attend

*“Now a presumptive maxima of 5 sessions* (*half days*) *unless higher figure justified on safeguarding grounds eg at risk and no one at home during day.”*

• Only allowing access to people who have critical or substantial need, according to the Fair Access to Care Services criteria

“People do need to have a greater need than social isolation which we used to be able to use.”

“Only those assessed as having complex and or multiple physical / learning disability now eligible for service.”

• Prohibiting people in residential homes from using the service

*“All service users in residential care are no longer receiving day services* (*due to double funding*), *and no new referrals have been taken in last 2 years so numbers have decreased.”*

*“Those in residential housing can no longer access day services*, *no matter how long they have attended and what they may have gained. They cannot choose to spend their day in a service out of their residential provider.”*

• Limiting access for self-funders

*“Those that are self-funding*, *even if they meet the criteria*, *cannot access the service.”*

“Even if wanting to spend their own money unable to buy day service.”

• Not offering day centre places to young people making the transition to adult social care services

“No school leavers have come into day service for 3 years – school leavers are offered personal budgets- not directed towards day services.”

Changes in referral procedure were also cited as leading to reduced eligibility for day services:

“Some referrals are only for 6 weeks. Greater emphasis on reablement which may include service users being offered voluntary sector or independent sector provisions where previously they would have been eligible for Day Care Centre provision.”

In another case, people who were not attending regularly have had their entitlement to the service withdrawn:

*“Elderly who have not attended for at least 4 to 6 weeks have been reassessed*, *roughly 98 per cent have not met the new eligibility criteria.”*

Not all respondents reported a narrowing of eligibility criteria. In one case, self-funders were to be able to attend local authority day centres for the first time, although the respondent noted: “Not sure how this will work in practice, or if they will want to use this service!”

Two-thirds of survey respondents indicated that charges had been introduced or increased for those using the service. The costs were highly variable – in some cases it meant paying for tea and coffee or meals rather than getting them free. However most respondents indicated large cost rises, from a nominal fee to a full-economic cost – for example, from a minimal charge to £50 a day – which would be deducted from the personal budget of anyone eligible for local authority funding.

*“Currently around £10 per day max*, *in future max will be actual cost of service* (*at least £48 per day*), *more for people with complexity issues subject to cap of disposable income.”*

*“Those service users who have been deemed as able to pay*, *have had to choose to not come because they can’t afford it or restrict their days to only one because of over-extortionate rises. Also those in receipt of benefits are also being restricted to only one - two days. Service users keep having letters sent regularly about charges to them.”*

*“Day services are now called ‘day opportunities’ and a budget of £30 provided. Effectively*, *this rations the service and means that someone can have only one day at a day centre funded by social services.”*

Service users with personal budgets are expected to have funds to cover these costs. However several respondents expressed concerns that not enough money was being made available in personal budgets to fund more than essential personal care services. As one put it,

“In my particular department most of the people I support only paid a minimal day charge. This will be increasing to approximately £50 a day. Most people cannot afford this and so will need to finish their placement in order to continue receiving essential care at home.”

The introduction of transport and meal charges were also mentioned by a number of people, along with increased use of pre-prepared meals.

There was a recurrent perception among some respondents that personal budgets and the broader personalization agenda were being used by local authorities as cover for cost-cutting exercises:

“It has given management the excuse to close Day Centres despite the fact that the number of service users wanting to use them has not dropped.”

“County Council say with personal budgets comes choice so service users need a variety of options. Smokescreen for cuts and closures in our view.”

*“PBs provides the overall ideological cover for reduction in services as in-house painted as unresponsive to needs*, *though no evidence for this as was uncovered during local campaigns.”*

The survey findings suggest that there are reasons to be concerned about the supply of building-based services in a context of personal budgets. Rather than day centre closures being the result of individuals making new commissioning decisions, the approach to day centres identified here has been one of local authorities *decommissioning* day centres and narrowing the choices on offer to people. The findings are not representative, but they do support other qualitative and quantitative studies in confirming the lack of attention to the supply of collective spaces in an era of personal budgets [36-38]. Based on a study of how self-directed support was shifting care provision in one local authority, Roulstone and Morgan warned of the shift from an ‘enforced collectivity’ of day services to an ‘enforced individualism’ of isolated individuals. Their findings – that ‘a great deal of former centre-based time was beginning to be spent at home’ [37][p.342] – resonates with the points made by survey respondents, half of whom did not know of any alternative provision for people whose day centre had closed.

### Conclusion

The three claims made in the personalization literature about how personal budgets would change individual, group and collective commissioning of services, have been interrogated through a focus on the national evaluation literature and a survey of staff working in day centres. The existing evaluation data indicates that individuals welcome the scope to make choices through being given a direct payment. However the limits of what the market can offer are evident, at the level of individual purchasing decisions, group pooling of budgets, and the funding of collective services. Complexity is particularly an issue in relation to individual and group purchasing, with uncertainty around what can and cannot be purchased and how to deal with employment issues. Local authorities have not delivered on the early promise that personalization would generate greater transparency and fewer rules on how money could be spent. Organisations such as TLAP, In Control and ADASS continue to campaign for transparent and fair allocation systems that give budget-holders maximum choice and control.

The data presented here draws on evaluation data and survey material which may not be representative of the experience of the majority of people who have now taken up personal budgets. Independent evaluation is required to confirm whether the potential dangers of individualised commissioning identified here are being realised as personal budget-holding grows. However there is enough material here to recognise that the co-occurrence of major funding cuts and a move to individualise the commissioning process creates a high risk that collective, building-based services will be undersupplied. The day centre closures identified by the survey seemed to have been triggered by local authority *decommissioning* rather than being a response to new commissioning choices by individuals acting as market actors. For all the optimism about personalization opening up choice and control for budget-holders, there is a lack of attention to the infrastructure which is required to enable alternative shared spaces to develop. As Beresford puts it: ‘There is an anxiety that the traditional menu of collective social care services – such as day centres and respite care - will wither away, leaving people adrift in a complex and inadequately regulated market: existing collective services may be closed without adequate alternative support provision being offered in replacement’ [36][p.12].

The limits of the market in responding to collective need and sustaining public spaces in which people can meet and support each other are well-rehearsed and underpinned the creation of many civic amenities, including day centres. Such an awareness does not obviate the need for social care support that is sensitive and responsive to the preferences of individuals. However it reaffirms the need for collective spaces in which people can share concerns and articulate forms of personalization based on inclusion and empowerment rather than isolation and risk-transfer.

## Competing interests

None.

## Declarations

Publication of this supplement was funded by the Nuffield Trust.

This article has been published as part of *BMC Health Services Research* Volume 13 Supplement 1, 2013: The Limits of Market-based Reforms. The full contents of the supplement are available online at http://www.biomedcentral.com/bmchealthservres/supplements/13/S1.
